# Management of sustainable urban green spaces through machine learning–supported MCDM and GIS integration

**DOI:** 10.1007/s11356-025-36367-7

**Published:** 2025-04-12

**Authors:** Murat Başeğmez, Ayhan Doğan, Cevdet Coşkun Aydın

**Affiliations:** 1 Department of Geographic Information System and Real Estate, Ministry of National Education, 06560 Beşevler, Ankara, Türkiye; 2 Department of Computer Technologies, Başkent OSB Vocational Higher School of Technical Sciences, Hacettepe University, 06909 Sincan Ankara, Türkiye; 3 Department of Geomatics Engineering, Hacettepe University, 06800 Beytepe, Ankara, Türkiye

**Keywords:** Analytic hierarchy process, Machine learning, Weighted linear combination, Technique for order preference by similarity to ideal solution, Geographic information systems, Green space suitability

## Abstract

This study evaluates green space suitability in İzmir’s Konak district using the analytic hierarchy process, machine learning, weighted linear combination, and the technique for order preference by similarity to ideal solution methods, integrated with geographic information systems. The approach enhances reliability in green space identification by ensuring consistent integration of weights determined by different methods. Machine learning enables dynamic adjustments to criterion weights, yielding the best results with the random forest algorithm. The analysis revealed that 75% of green spaces were sub-optimally located, with optimal zones in the western and northern regions. The technique for order preference by similarity to ideal solution methodology prioritized area ZGS_6 as the most suitable area, while ZGS_4 ranked lowest. This method supports efficient resource allocation and improves budgeting processes. Hence, the integration of multi-criteria decision-making and machine learning with geographic information systems enhances the planning of sustainable cities and offers critical insights to decision-makers who prioritize sustainability and livability.

## Introduction

Throughout history, cities have existed as areas inhabited by a small portion of the population. However, with the technological and economic developments that began in the eighteenth century, cities expanded rapidly and became centers of attraction and migration. The swift advancement of technology has led to increased demands for health, employment, education, and comfortable living, which has resulted in significant urban population growth. According to a report published by the United Nations, the world population is expected to reach 8.5 billion by 2030, 9.7 billion by 2050, and 10.4 billion by 2100 (United Nations 2023). Consequently, this projected increase may lead to a proportional rise in population density within cities.

Considering current and future population projections, it is anticipated that challenges in areas such as infrastructure, transportation, environment, education, health, and employment will continue to escalate in urban settings. Therefore, to enhance livability and ensure sustainability, local administrators and policymakers need to prioritize green spaces. Globally, various strategies and studies are being developed for the planning and expansion of green areas to reduce environmental risks and increase livable spaces (Dimitrova and Dzhambov 2017; Nowak et al. 2018; Wu et al. 2019). Although these efforts aim to improve the livable and sustainable structures of cities, there are several challenges encumbering the process. The provision of green spaces, which is an important reference point for people and an integral part of environmental planning, poses certain challenges for planners and local governments. These challenges include criteria such as topography, walkability, proximity to residences, and access to main roads, all of which have a significant impact on the design of green spaces (Homes and Communities Agency 2013). Despite these challenges, the green spaces that are planned according to the specified criteria are of critical importance in reducing environmental problems, improving people’s health and living conditions, and thus contributing to sustainable urban models. Additionally, green spaces play a significant role in enhancing air quality, regulating temperatures, and mitigating the adverse effects of climate change in cities. Thus, urban green spaces contribute to biodiversity not only through carbon sequestration but also by providing habitat fragments and stepping stones for species (Aronson et al. 2017; Liu and Russo 2021; Jabbar et al. 2022; Hu and Lima 2024).

In Türkiye, for instance, the “Eleventh Development Plan (2019–2023)” and the “Twelfth Development Plan (2024–2028)” have been prepared with the aim of creating sustainable cities and enhancing livability. The Eleventh Development Plan, for instance, is aimed at establishing accessible and highly connected transportation systems, creating infrastructure resistant to disasters and climate change, and developing long-term integrated urban plans. In this context, it is aimed to increase green spaces such as “National Gardens” and ensure walkability in these areas. Moreover, disaster hazard and risk maps have been prepared by considering scenarios related to the effects of climate change in tandem with measures to improve the quality of urban life (Presidency of the Republic of Türkiye 2019). On the other hand, the Twelfth Development Plan is proposed to facilitate the accessibility of green spaces and plan them by taking into account design criteria based on city size, population density, accessibility, climate, and geographical conditions (Presidency of the Republic of Türkiye 2023).

Given the benefits that green spaces provide to cities and their inhabitants, it is of great importance for urban planning and sustainability to identify these areas in suitable locations. For instance, studies conducted worldwide recommend providing at least 10 m^2^ of green space per capita in planning regions (El Amraoui et al. 2018; Pokhrel 2019). Additionally, the criteria used in the selection of green spaces include land use, elevation, slope, distance to wetlands, proximity to main roads, and proximity to urban centers (Osséni et al. 2024). In addition to the above-mentioned criteria, factors such as area size, transportation, access distance to green spaces, zoning plans, proximity to health institutions, security centers, educational areas, markets, and parking lots are actively considered in site selection (Kuşkonmaz 2020; Topçu et al. 2023).

To effectively solve environmental and planning-related problems in cities, it is necessary to manage spatial and attribute data of cities systematically and efficiently. In this context, the integration of MCDM (multi-criteria decision making) and GIS (geographic information systems) methods provides multidimensional contributions to urban planning and development. For instance, in a study conducted in Debre Markos City, multi-criteria analyses were utilized in conjunction with remote sensing and GIS technologies to evaluate existing and proposed urban green spaces (Anteneh et al. 2023). This approach demonstrated significant potential for enhancing residents’ quality of life. Another study in Liverpool reclassified the types and functions of green infrastructure, thereby providing critical data that local authorities can utilize when preparing spatial plans (Kim and Min 2024). Similarly, Amsterdam’s participatory GIS model has created an important link in urban greening efforts by integrating data-driven approaches with citizen-centric policies (Mattijssen et al. 2024).

Moreover, the connection between green infrastructure and atmospheric conditions highlights the economic value of such infrastructure. For instance, it has been observed that homebuyers are willing to pay higher prices for properties located in areas with well-developed green infrastructure. However, the decision-making processes that lead to these price increases are not yet fully understood (Zalejska-Jonsson et al. 2020). A study conducted in Fuzhou City developed a comprehensive model by integrating landscape ecology and spatial syntax theories with GIS technologies to examine the spatial accessibility of green spaces (Huang et al. 2023). This model has provided both theoretical and technical support for ecological function planning and sustainable development.

In addition to these methods, machine learning (ML) maximizes environmental benefits by offering innovative solutions for the design and maintenance of urban green spaces. With its capacity to analyze and model the complex relationships within urban ecosystems, ML significantly contributes to urban planning. For instance, by utilizing ML techniques, the spatial structure of green spaces can be optimized, thereby reducing PM2.5 concentrations and land surface temperatures (Li et al., 2023; Nizamani et al., 2024). Moreover, ML models can evaluate factors such as waste levels, maintenance conditions, and pollution, thereby enhancing green space quality and improving public perception (Vineela et al. 2023). In addition to these applications, ML provides valuable insights into urban planning by uncovering relationships between urban blue-green spaces and carbon sequestration (Wu et al., 2024).

Thus, this study aims to evaluate the site selection and suitability of existing green spaces in light of global and national research. The Konak district of İzmir was selected as the study area, and the requisite data for the evaluation of green spaces were digitized. Therefore, using the database created from the obtained data, a green space suitability assessment of the study area was conducted within a GIS environment. The weights for each criterion were determined using the AHP (analytic hierarchy process) and ML methods, and these weights were subsequently integrated into the map layers of each criterion using the WLC (weighted linear combination) method. Consequently, decision maps with a cell resolution of 30 m × 30 m were created, and the suitability of existing green spaces was evaluated. Finally, the TOPSIS (technique for order preference by similarity to ideal solution methods) method was used to identify new investment areas.

## Material and methods

### Study area

**Fig. 1:**
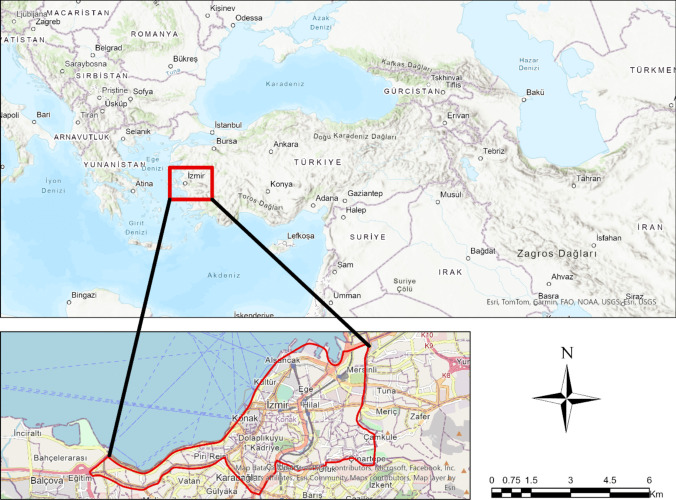
Study area

To determine suitable locations for green spaces and evaluate the suitability of existing ones, the Konak district of İzmir (27.134898°E, 38.428673°N) was selected as the study area (Fig. 1). This area was chosen due to its central location and the fact that 7.31% of İzmir’s population resides there. Additionally, Konak is the fifth most populous district of İzmir with a population of 327,300, according to 2024 data from the Turkish Statistical Institute (Turkish Statistical Institute (TSI) 2024).

### Data

The criteria used in this study (Table 1) were identified by reviewing national and international publications on green space site selection, as well as by consulting local experts (e.g., urban planners, architects, civil engineers, and geomatic engineers). These experts provided insights into the local context, feasibility, and priority levels for each criterion.

**Table 1:** Data layers

Data layer name	Data type	Date	Data source
Subway station	Point	2024	(Izmir Metropolitan Municipality (IMM) 2024)
Bus station	Point	2024	IMM, 2024
Railway station	Point	2024	IMM, 2024
Bike sharing station	Point	2024	IMM, 2024
Parking	Point	2024	IMM 2024
Slope	Raster	2024	(National Aeronautics and Space Administration (NASA) 2024)
Religious area	Point	2024	IMM, 2024
Public sector area	Point	2024	IMM, 2024
School	Polygon	2024	(Ministry of National Education (MoNE) 2024)
Health facility	Point	2024	IMM, 2024
Sports facility	Polygon	2024	(Izmir Metropolitan Municipality (IMM) 2024)
Cycling path	Line	2024	IMM, 2024)
Railway	Line	2024	IMM, 2024
Subway	Line	2024	IMM, 2024
Main road	Line	2024	IMM, 2024

After identifying these data layers, scores were established for each criterion based on a combination of a literature review and expert input. Table 2 presents the final scoring used in the spatial analyses. Once all scores were determined with a literature review, each dataset was projected into the World Geodetic System (WGS) 1984 datum and then transformed to the Universal Transverse Mercator (UTM) coordinate system, Zone 35 N, to ensure spatial consistency across all layers.

**Table 2:** Criteria and sub-criteria

Criteria	Sub-criteria	Score
Subway station	< 250 m 250–500 500–750 750–1000 1000 <	5 4 3 2 1
Bus station	< 250 m 250–500 500–750 750–1000 1000 <	5 4 3 2 1
Railway station	< 250 m 250–500 500–750 750–1000 1000 <	5 4 3 2 1
Bike sharing station	< 250 m 250–500 500–750 750–1000 1000 <	5 4 3 2 1
Parking	< 100 m 100–200 200–300 300–400 400 <	5 4 3 2 1
Slope	< 5° 5–10 10–15 15–20 20 <	5 4 3 2 1
Religious area	< 250 m 250–500 500–750 750–1000 1000 <	5 4 3 2 1
Public sector area	< 500 m 500–1000 1000–1500 1500–2000 2000 <	5 4 3 2 1
School	< 500 m 500–1000 1000–1500 1500–2000 2000 <	5 4 3 2 1
Health facility	< 500 m 500–1000 1000–1500 1500–2000 2000 <	5 4 3 2 1
Sports facilities	< 250 m 250–500 500–750 750–1000 1000 <	5 4 3 2 1
Cycling path	< 250 m 250–500 500–750 750–1000 1000 <	5 4 3 2 1
Railway	< 250 m 250–500 500–750 750–1000 1000 <	5 4 3 2 1
Subway	< 250 m 250–500 500–750 750–1000 1000 <	5 4 3 2 1
Main road	< 50 m 50–100 100–150 150–200 200 <	5 4 3 2 1

### WLC

Among MCDM methods, WLC is one of the most widely used techniques. This method involves multiplying the values of the criteria by the weights assigned by decision-makers and then summing these products to obtain a final score. The mathematical expression of the method is presented below (Hwang and Yoon 1981; Kim and De Weck 2006).

1 $$\begin{array}{c}\begin{array}{c}A(i)=\sum\nolimits_{j=i}^na\left(i,j\right)w\left(j\right)i=1,2,3,\dots\\A(i):\;Weighted\;total\;score\;of\;alternatives\;(i)\\A(i,j):\;Score\;of\;alternative\;(i)\;according\;to\;(j)\;criteria\\W(j):\;Weight\;of\;j\;criteria\end{array}\end{array}$$

### AHP

AHP, developed by Thomas L. Saaty, is one of the most widely used MCDM methods. The method provides solutions by employing pairwise comparisons within multi-level hierarchical structures (Saaty 2008). In alignment with the defined objectives, alternatives are determined by ranking criteria and sub-criteria. During this process, decision-makers ascertain the relative importance of the criteria through pairwise comparison matrices. These importance values are determined using the scales provided by AHP (Ma et al. 2005), which ensures a homogeneous determination of criterion weights. After constructing the pairwise comparison matrices based on the criteria, the criterion weights are calculated.

### Machine learning

**Fig. 2:**
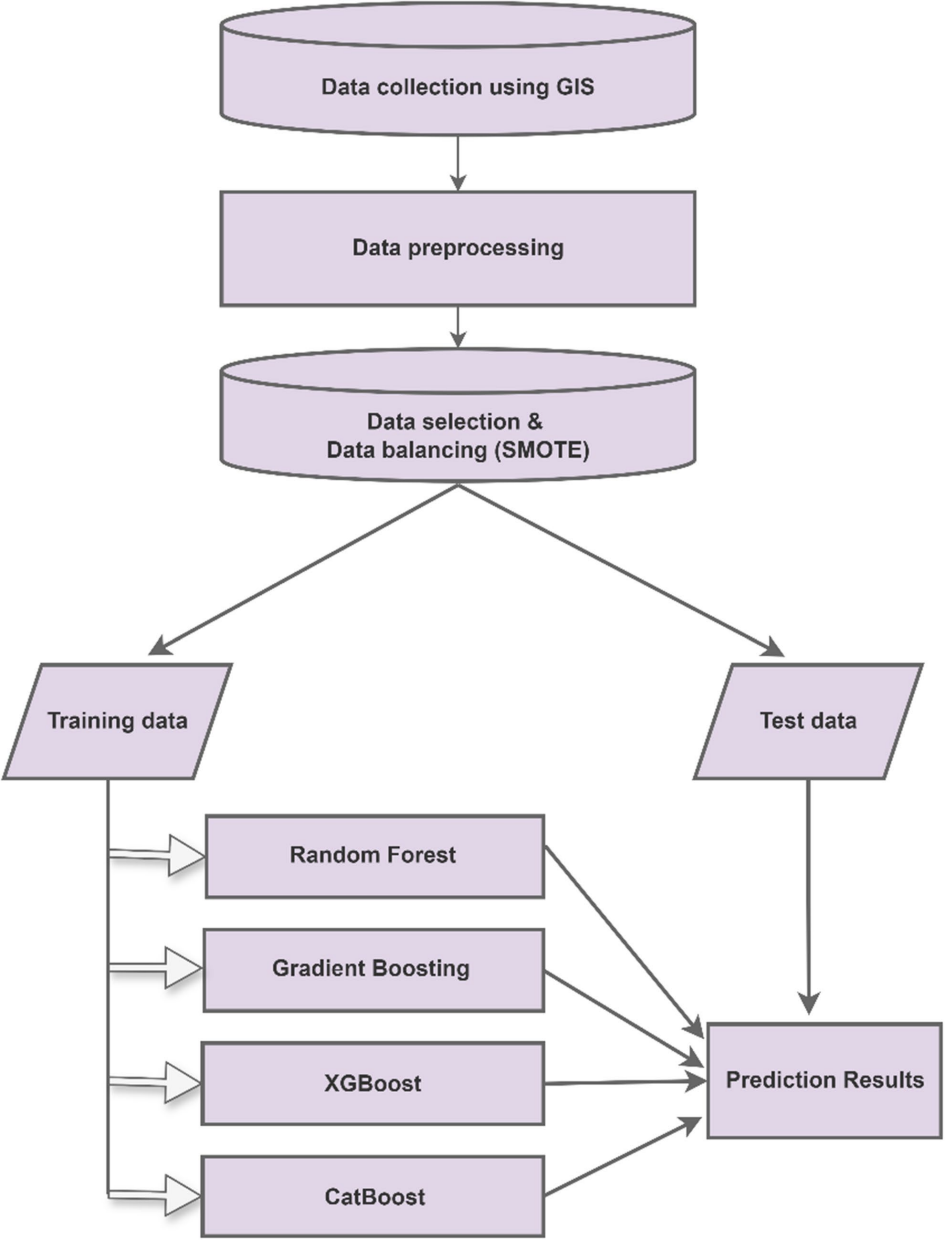
The workflow diagram of the ML

ML consists of artificial intelligence methods that enable computers to learn to use various techniques and algorithms. ML constructs the necessary models to perform the desired tasks by analyzing datasets of varying sizes, and predictions are made using these models. Numerous algorithms are utilized in ML processes. In this study, methods such as random forest (RF), gradient boosting (GB), extreme gradient boosting (XGBoost), and category boosting (CatBoost) were employed. The workflow diagram of the ML processes is presented in Fig. 2.

In this study, the data used for the ML processes were obtained using the GIS software. After preprocessing the data, the synthetic minority oversampling technique (SMOTE) was employed to address the issues arising from the imbalanced data classes (Chawla et al. 2002). To validate the model performance, the *K*-fold cross-validation method was utilized (Jiang and Chen 2016; Zhang and Liu 2023). Thereafter, the results were evaluated using metrics such as accuracy, precision, recall, and the F1 score.

#### Random forest method

RF is an ensemble learning method widely used to solve various problems. It makes predictions by aggregating the outputs of multiple decision trees, with the final result obtained through majority voting or by averaging the predictions of each tree. RF is popular due to its high accuracy and its ability to minimize overfitting (Breiman 2001).

To achieve these advantages, RF constructs decision trees by randomly selecting subsets of the training data. Each tree is trained on a different subset, which enhances the generalization ability of the model. Additionally, during the construction of each tree, only a randomly selected subset of features is considered at each split. This approach increases diversity among the trees and results in a stronger and more robust model (Liaw 2002).

Owing to its robustness and accuracy, RF has been successfully applied across various fields and datasets. For instance, it has been used in disease prediction in healthcare, credit risk analysis in the financial sector, and earthquake prediction (Doğan 2024). Its strong performance, especially on complex data structures and large datasets, makes this method particularly appealing (Cutler et al. 2007).

#### Gradient boosting method

GB is a powerful ensemble method widely used in statistical learning and ML. This approach typically makes predictions by constructing a series of weak learners, such as decision trees, where each new tree aims to reduce the error rates of the previous ones. GB is an iteratively optimized method designed to enhance model accuracy; at each iteration, it attempts to minimize errors, thereby reducing the overall loss function (Friedman 2001).

In this methodology, the error rate of each weak learner is corrected using the logic of backpropagation. Specifically, each new tree focuses on learning the residual errors of the existing model. This iterative process results in an optimized model that improves prediction accuracy. One of the key advantages of GB is its high performance on complex datasets and its effectiveness across various domains, such as financial forecasting, earthquake prediction, and health analysis (Hastie et al. 2009; Doğan 2023).

There are several variations of the GB method. Among these are popular algorithms like XGBoost and LightGBM, which offer additional optimizations and acceleration techniques to enhance performance. Such variations provide significant advantages, especially when dealing with large datasets and complex models (Chen and Guestrin 2016; Ke et al. 2017).

#### Category boosting method

CatBoost is a machine learning method specifically developed to work effectively with categorical data. Traditional boosting algorithms, which generally rely on numerical data, often struggle when interacting with categorical variables. To address this issue, CatBoost focuses on the efficient processing of categorical features (Prokhorenkova et al. 2018).

To achieve this, CatBoost constructs a more general structure that represents all categorical values, rather than creating separate trees for each unique value in the categorical data. This approach allows the model to enhance its overall performance by considering the attributes of each category. Thus, CatBoost strengthens the model’s generalization capabilities by effectively handling the complex structure of categorical data, which typically requires extensive feature engineering.

As a result of these advantages, CatBoost demonstrates high performance, especially on large datasets and situations involving numerous categorical variables. It has been applied in various fields such as healthcare, earthquake prediction, and marketing (Doğan 2024). Improved management of categorical data significantly increases the predictive power and accuracy of the model (Gupta et al. 2021).

#### Extreme gradient boosting method

The XGBoost algorithm is an enhanced version of the gradient boosting algorithm, which is known for its high performance on large datasets and complex models. By focusing on speed and efficiency, XGBoost offers faster model training compared to standard boosting methods. This algorithm provides strong predictive performance in both regression and classification problems (Chen and Guestrin 2016).

In addition to its speed advantages, XGBoost also incorporates regularization techniques that help reduce the risk of overfitting. Its parallel computing capabilities allow for faster model training across multiple processors. When constructing each decision tree, XGBoost aims to minimize the errors of the preceding trees as much as possible. This iterative process enhances model accuracy while enabling rapid and efficient computation (Friedman 2001; Nadar 2023).

Due to these strengths, XGBoost has been applied in various fields such as healthcare, finance, and marketing and is frequently preferred in data science competitions like Kaggle. Its high accuracy and speed in classification and regression tasks have made it a popular choice among researchers and data scientists (Zhang et al. 2022; Doğan 2023).

### TOPSIS

TOPSIS is an effective and widely used method among MCDM techniques that facilitates the objective ranking of alternatives in decision-making processes. The primary aim of this method is to assist decision-makers in identifying the most suitable option among a set of alternatives. TOPSIS evaluates the relative performance of each alternative by calculating its proximity to the ideal (positive) solution and its distance from the worst (negative) solution, thereby simplifying the determination of the most appropriate choices (Yoon and Hwang 1981).

One of the main advantages of TOPSIS is that it can provide an objective assessment based on both ideal and worst-case scenarios. Additionally, it is easily applicable in multi-criteria decision-making processes and offers a systematic approach (Yoon and Hwang 1995). TOPSIS also enables the comparison of alternatives against each other, thus allowing decision-makers to make more informed and balanced choices. However, the TOPSIS method has certain limitations. For example, the assumption of independence among criteria may not always hold true, and the process of accurately determining the weights of criteria can be subjective (Yoon and Hwang 1981). Moreover, errors made during the identification of ideal and negative ideal solutions can negatively impact the accuracy of the results.

### Integration of AHP, ML, and GIS for green space suitability assessment

The integration of AHP, machine learning, and GIS in this study offers a comprehensive and systematic approach to assessing the suitability of urban green spaces. This methodological framework was designed to address the limitations of traditional decision-making models by combining expert-driven assessments with data-driven insights while ensuring spatial applicability. The choice of this hybrid approach over alternative methods, such as fuzzy AHP and entropy weighting, is based on its ability to incorporate both qualitative expert judgments and quantitative data analysis, ultimately enhancing the accuracy and objectivity of the assessment process. AHP was selected for its structured pairwise comparison methodology, which allows for a systematic evaluation of decision criteria based on expert opinions. Its hierarchical structure ensures that complex multi-criteria decision-making problems can be broken down into a logical framework, facilitating a more transparent and rational decision-making process. One of the critical advantages of AHP is its ability to incorporate expert knowledge into the weighting process while maintaining consistency validation through the calculation of the consistency ratio (CR). This ensures that expert judgments are not only subjective opinions but follow a structured and rational framework. However, a major limitation of AHP is its dependence on subjective expert input, which can introduce bias, especially in large-scale spatial analyses. To address this limitation, machine learning methods were incorporated into the study, which allowed for a more objective and data-driven approach to weighting. The use of RF, XGBoost, CatBoost, and GB facilitated the extraction of patterns from existing urban green space distributions and enabled the identification of key factors influencing suitability. Unlike AHP, which relies on expert intuition, ML algorithms derive weights by analyzing large datasets, recognizing nonlinear relationships, and detecting complex spatial dependencies. This data-driven approach minimizes subjectivity and enhances the reliability of the weighting process, particularly in dynamic urban environments where multiple interacting factors influence decision-making.

Incorporating GIS into this framework was essential for the spatial integration and analysis of the weighted criteria. GIS facilitated the WLC method, which allowed for the overlay and aggregation of multiple spatial layers based on their respective weights. This process ensured that the final suitability maps were not only grounded in expert evaluations and data-driven insights but also spatially coherent, thereby reflecting real-world urban planning constraints and opportunities. The precision of the spatial analysis was enhanced using raster data with a resolution of 30 m × 30 m, which made it possible to evaluate the suitability of green spaces across the study area in high resolution.

The superiority of this hybrid AHP-ML-GIS approach over alternative methodologies is evident in its ability to balance expert knowledge with empirical data while maintaining spatial integrity. Compared to fuzzy AHP, which introduces uncertainty modeling into expert judgments but remains heavily reliant on subjective input, the hybrid model leverages ML to incorporate real-world spatial patterns, which reduces reliance on purely theoretical decision-making. Similarly, entropy weighting, while effective in identifying data variability, lacks the ability to integrate expert knowledge, making it less suitable for applications requiring domain-specific insights. The proposed hybrid approach, therefore, offers a more robust, adaptable, and scientifically rigorous methodology that enhances the accuracy and applicability of urban green space assessments.

To ensure a balanced and reliable decision-making process, the final hybrid weighting method was developed by multiplying the AHP-derived weights with the RF-derived weights and subsequently normalizing them. This process ensured that the final criterion weights incorporated both expert knowledge and data-driven insights and effectively addressed the limitations of individual methodologies. The spatial integration of these weighted criteria in GIS facilitated the generation of high-resolution suitability maps, which were then analyzed using the TOPSIS method to rank the most suitable locations for green space development. By applying multi-criteria decision-making, machine learning, and spatial analysis techniques in a fully integrated manner, the study provides an advanced methodological framework that can be effectively used in sustainable urban planning.

This integration of AHP, ML, and GIS not only enhances the scientific rigor of the study but also improves its practical applicability by providing policymakers and urban planners with a robust decision-support system. The methodology ensures that urban green space planning is based on both expert judgment and empirical spatial analysis, which ultimately leads to more sustainable and well-informed planning decisions. By leveraging expert-driven, data-driven, and spatially aware decision-making tools, this study offers a novel contribution to the field of urban sustainability assessment and provides a replicable and scalable approach for future research and policy applications.

### Data processing and preprocessing methods

To ensure the accuracy and consistency of the data used in this study, a structured data processing workflow was implemented. The dataset consists of spatial layers obtained from multiple sources, including the Izmir Metropolitan Municipality (IMM), the Turkish Statistical Institute (TSI), the Ministry of National Education (MoNE), and NASA. All datasets represent the most recent available data as of 2024, thereby ensuring that the analysis reflects the current urban conditions and planning requirements. While most datasets were directly acquired or digitized, the slope data were derived from a digital elevation model (DEM) obtained from NASA. This DEM dataset was processed in ArcGIS Pro using the Spatial Analyst Toolset, where the slope function was applied to compute terrain slopes from elevation values. This produced a continuous raster representation of slope variations across the study area.

To integrate and standardize different data formats, all vector layers (points, lines, and polygons) were processed according to predefined subcategories. A buffering operation was applied to relevant vector features, such as subway stations, bus stops, and public sector areas, to classify proximity-based spatial relationships. The buffered features were then stored in a geodatabase and subsequently converted into raster format using the ArcGIS Pro to ensure compatibility with raster-based spatial analysis methods. Once rasterization was completed, all datasets were standardized to a 30 × 30-m spatial resolution to optimize the balance between computational efficiency and spatial accuracy.

Following this, a reclassification process was performed using the Reclassify Tool in ArcGIS Pro to align the data values with the standardized classification scheme defined in Table 3. This transformation enabled a structured and uniform representation of all criteria, as well as a subsequent multi-criteria evaluation and spatial overlay analysis. The systematic preprocessing of the datasets enhanced the robustness and reproducibility of the study’s methodological framework by ensuring that all spatial layers were correctly aligned, standardized, and fully prepared for analytical integration.

**Table 3:** The weights of criteria (AHP)

Criteria	W
Subway station	0.0950
Bus station	0.0942
Railway station	0.0485
Bike sharing station	0.0132
Parking	0.0355
Slope	0.1879
Religious area	0.0409
Public sector area	0.0409
School	0.0797
Health facility	0.0788
Sports facility	0.0546
Cycling path	0.0179
Railway	0.0378
Subway	0.0534
Main road	0.1217

## Application stage

In this section, the weights of the identified criteria were calculated using both AHP and ML methods. The obtained weight values were multiplied to determine the final weights. Subsequently, the WLC method was applied, wherein each criterion’s thematic map layers were associated with these weights to create a decision map. The resulting decision map was then divided into five classes to identify suitability zones for green spaces. The most suitable regions within each area were then prioritized using the TOPSIS method. This methodological approach contributes to the decision-making process by providing a systematic and objective evaluation for the optimal selection of green spaces.

### Determination of criteria weights with AHP

To determine the weights of the criteria using the AHP methodology, a survey was conducted among 20 experts with diverse professional backgrounds to ensure a broad perspective. Specifically, five urban planners, six geomatics (surveying) engineers, five architects, and four civil engineers participated in the study. The experts were selected based on their experience in urban development, green space design, and infrastructure projects.

During the survey, participants were asked to rate the relative priorities of the criteria on a 1 to 9 scale (1 = equally important; 9 = extremely more important) to construct a pairwise comparison matrix. Each expert completed an individual questionnaire, and the responses were then aggregated to form a single comparison matrix. Following the standard AHP procedure, the consistency ratio (CR) was calculated to ensure reliability; a CR value below 0.10 indicates acceptable consistency. In this study, the CR was found to be 0.089, confirming that the experts’ judgments were sufficiently coherent.

Table 3 presents the final weights of the criteria derived from the aggregated pairwise comparison matrix. These weights reflect the collective expert opinion on the importance of each criterion for urban green space site selection.

### Determination of criteria weights with ML

To determine the weights using ML methods, the existing green spaces within the study area were first digitized. Subsequently, the class values for each corresponding classified data layer at these locations were identified. Similarly, points identified as potential green space locations within the study area were digitized, and their respective classified data layer class values were determined. Prior to executing the ML processes, the data were cleansed and transformed to fit a categorical structure suitable for ML applications. To address class imbalance within each category, SMOTE was employed (Chawla et al. 2002).

**Fig. 3:**
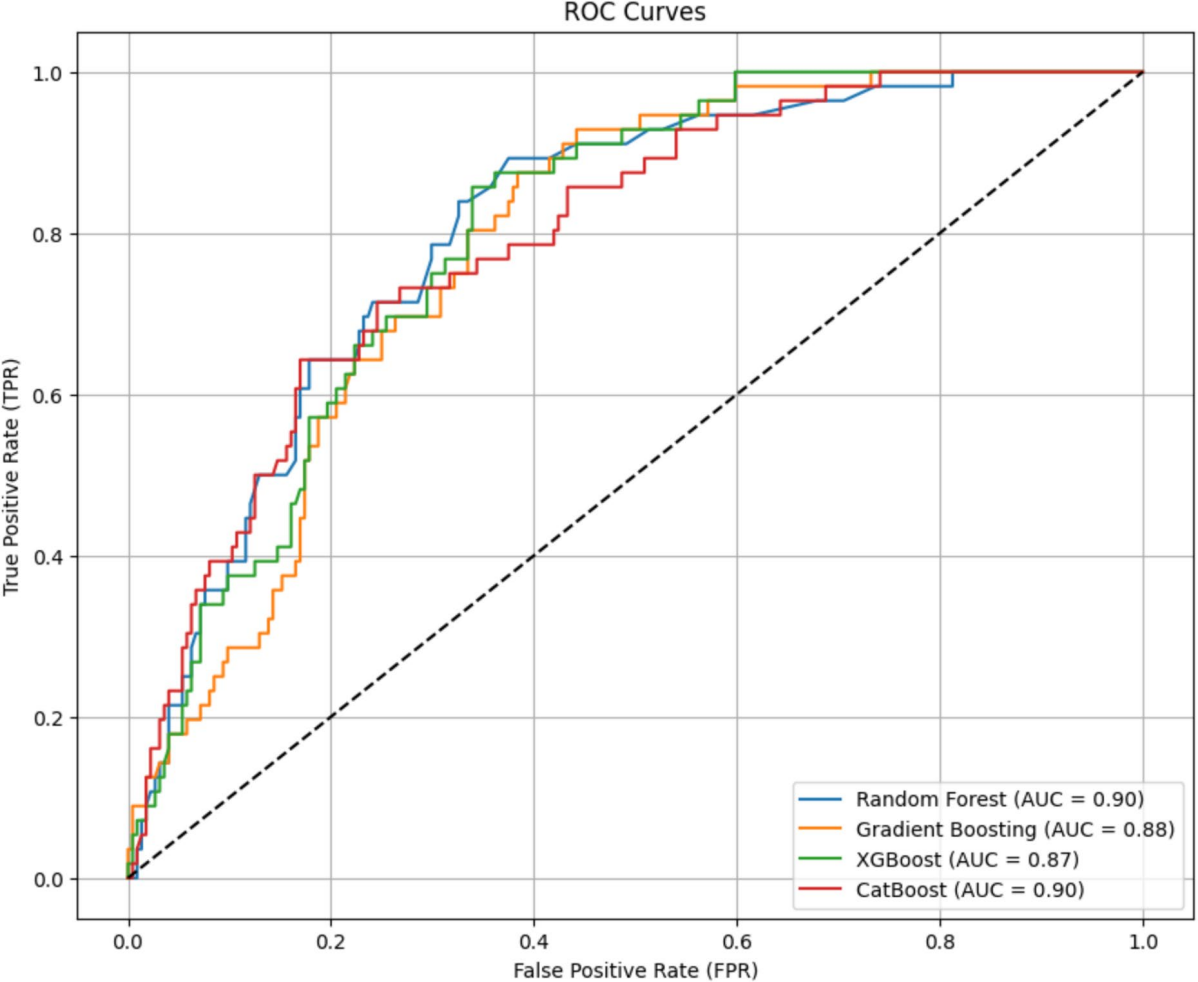
ROC curves and AUC values of ML methods

The model performance was validated using the five-fold *K*-fold cross-validation method (Jiang and Chen 2016; Zhang and Liu 2023). The implementation of data balancing and cross-validation techniques was intended to mitigate the risks of overfitting and underfitting. Subsequently, the models were trained and tested, and the performance of the ML methods was evaluated using metrics such as accuracy, precision, recall, and F1 score. These results are presented in Table 4. Additionally, the receiver operating characteristic (ROC) curves and the area under the curve (AUC) values for the ML methods are illustrated in Fig. 3.

**Table 4:** The metrics of ML methods

ML methods	Accuracy	Precision	Recall	F1 score
RF	0.69	0.70	0.69	0.68
GB	0.64	0.64	0.64	0.63
XGBoost	0.63	0.63	0.63	0.62
CatBoost	0.68	0.70	0.68	0.68

Furthermore, the weights of each criterion were determined based on their importance using ML methods such as CatBoost, GB, RF, and XGBoost (Table 5). This comprehensive approach ensures a systematic and objective evaluation of green space suitability, thereby enhancing the decision-making process for optimal green space selection.

**Table 5:** The weights of criteria

Criteria	CatBoost	GB	RF	XGBoost
Subway station	0.0512	0.0621	0.0639	0.0708
Bus station	0.0477	0.0446	0.0449	0.0321
Railway station	0.0670	0.0806	0.0743	0.0735
Bike sharing station	0.0428	0.0848	0.0706	0.1215
Parking	0.0542	0.0529	0.0645	0.1078
Slope	0.1064	0.0916	0.0930	0.0704
Religious area	0.0591	0.0492	0.0580	0.0905
Public sector area	0.0741	0.0540	0.0559	0.0565
School	0.0054	0.0153	0.0176	0.0312
Health facility	0.0673	0.0339	0.0504	0.0399
Sports facilities	0.0605	0.0992	0.0753	0.0909
Cycling path	0.0500	0.0656	0.0626	0.0715
Railway	0.1079	0.0957	0.0875	0.0398
Subway	0.0543	0.0515	0.0686	0.0527
Main road	0.1522	0.1189	0.1130	0.0508

The analysis revealed that both the RF and CatBoost methods outperformed the other approaches. However, when all the metrics were evaluated collectively, the RF method emerged as the most successful. Consequently, the criteria weights derived from the RF method were selected for incorporation into the hybrid approach.

Among the various machine learning (ML) models employed in this study, random forest (RF) was selected as the primary algorithm for determining criterion weights due to its superior performance in both classification accuracy and feature importance estimation. While XGBoost, CatBoost, and gradient boosting (GB) were also evaluated, RF demonstrated the most balanced and reliable performance across multiple evaluation metrics.

The comparative analysis of ML models (Table 4) reveals that RF achieved the highest levels of accuracy (0.69), precision (0.70), recall (0.69), and F1-score (0.68). Furthermore, RF attained an AUC value of 0.90, indicating superior model robustness compared to other boosting-based algorithms. These results suggest that RF is particularly effective in handling multi-dimensional spatial datasets and capturing the complex relationships between green space suitability factors.

In addition to its predictive accuracy, RF was chosen for its computational efficiency and reduced risk of overfitting, which makes it well-suited for applications involving large-scale urban spatial data. Unlike boosting methods such as XGBoost and CatBoost, which require extensive parameter tuning and are prone to overfitting in small-to-medium datasets, RF employs a bagging approach that enhances generalizability by averaging multiple decision trees. This characteristic ensures that the resulting criterion weights are not only data-driven but also robust against variations in input datasets.

Another key advantage of RF in this study is its feature importance evaluation capability, which enables transparent and interpretable weight determination. By leveraging RF’s ability to capture complex, nonlinear relationships in spatial data, this study enhances the accuracy and reliability of the weighting process, thereby ensuring that the final decision-support framework is both scientifically robust and practically applicable in urban green space planning.

To integrate RF-based criterion weights with AHP-derived weights, a hybrid weighting approach was applied. In this process, the criterion weights obtained from AHP (*W*_AHP_) and RF (*W*_RF_) were multiplied to generate a composite weight (*W*_Hybrid_) that balances expert judgment with data-driven insights:

2 $$W_{Hybrid}=W_{AHP}\times W_{RF}$$

 

Since this multiplication alters the original scale of the weights, normalization was performed to ensure that the final sum of the weights equals 1 and maintains relative consistency. The normalization process was carried out using the following equation:

3 $${W}_{\text{Normalized}}=\frac{{W}_{\text{Hybrid}}}{\sum {W}_{\text{Hybrid}}}$$

This normalization ensures that the combined weights remain proportionally accurate and prevents any single criterion from disproportionately influencing the final ranking. By applying this hybrid weighting method, the study effectively integrates qualitative expert-based knowledge (AHP) with quantitative data-driven learning (RF) to create a more robust and adaptive decision-making framework for the assessment of urban green space suitability.

### Determination of criteria weights with a hybrid method

A study similar to the proposed hybrid model was conducted using the fuzzy analytic hierarchy process (Fuzzy AHP) and RF methods (Mohsin et al. 2022). Building upon this previous work, the weights derived from the AHP and RF methods were multiplied to determine the final criterion weights for the proposed hybrid model. Subsequently, the obtained weights were normalized so that their total sum equals one (Table 6). This process established the criterion weights to be used in the implementation phase.

**Table 6:** The weights of criteria via the hybrid method

Criteria	AHP	RF	Hybrid method	Normalized hybrid method
Subway station	0.0512	0.0639	0.0033	0.0431
Bus station	0.0477	0.0449	0.0021	0.0282
Railway station	0.0670	0.0743	0.0050	0.0656
Bike sharing station	0.0428	0.0706	0.0030	0.0398
Parking	0.0542	0.0645	0.0035	0.0461
Slope	0.1064	0.0930	0.0099	0.1304
Religious area	0.0591	0.0580	0.0034	0.0452
Public sector area	0.0741	0.0559	0.0041	0.0546
School	0.0054	0.0176	0.0001	0.0013
Health facility	0.0673	0.0504	0.0034	0.0447
Sports facilities	0.0605	0.0753	0.0046	0.0600
Cycling path	0.0500	0.0626	0.0031	0.0412
Railway	0.1079	0.0875	0.0094	0.1244
Subway	0.0543	0.0686	0.0037	0.0491
Main road	0.1522	0.1130	0.0172	0.2266

### Determination of the most suitable green spaces with a hybrid method

**Fig. 4:**
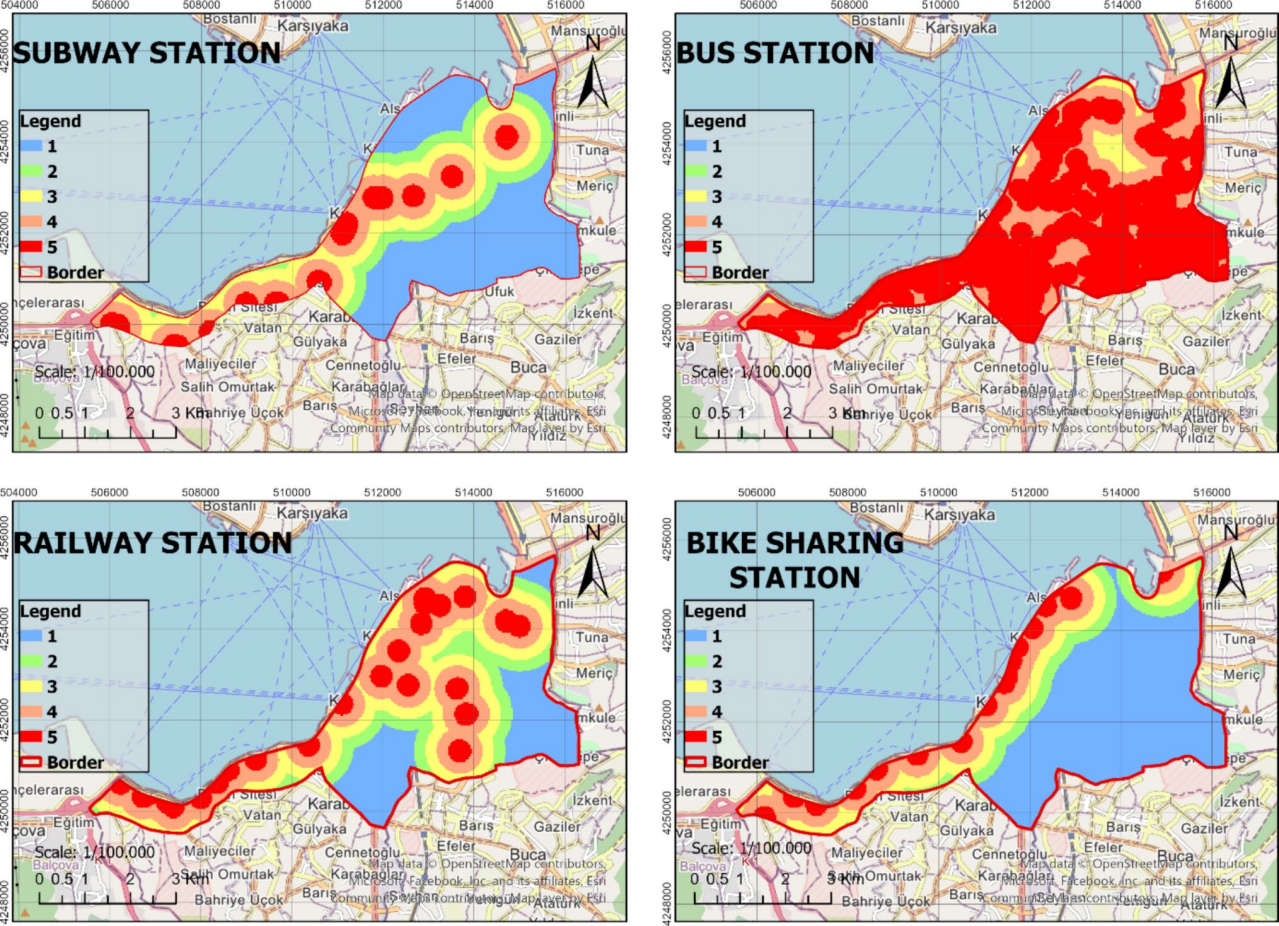
Suitable map (Criteria: 1–4)

**Fig. 5:**
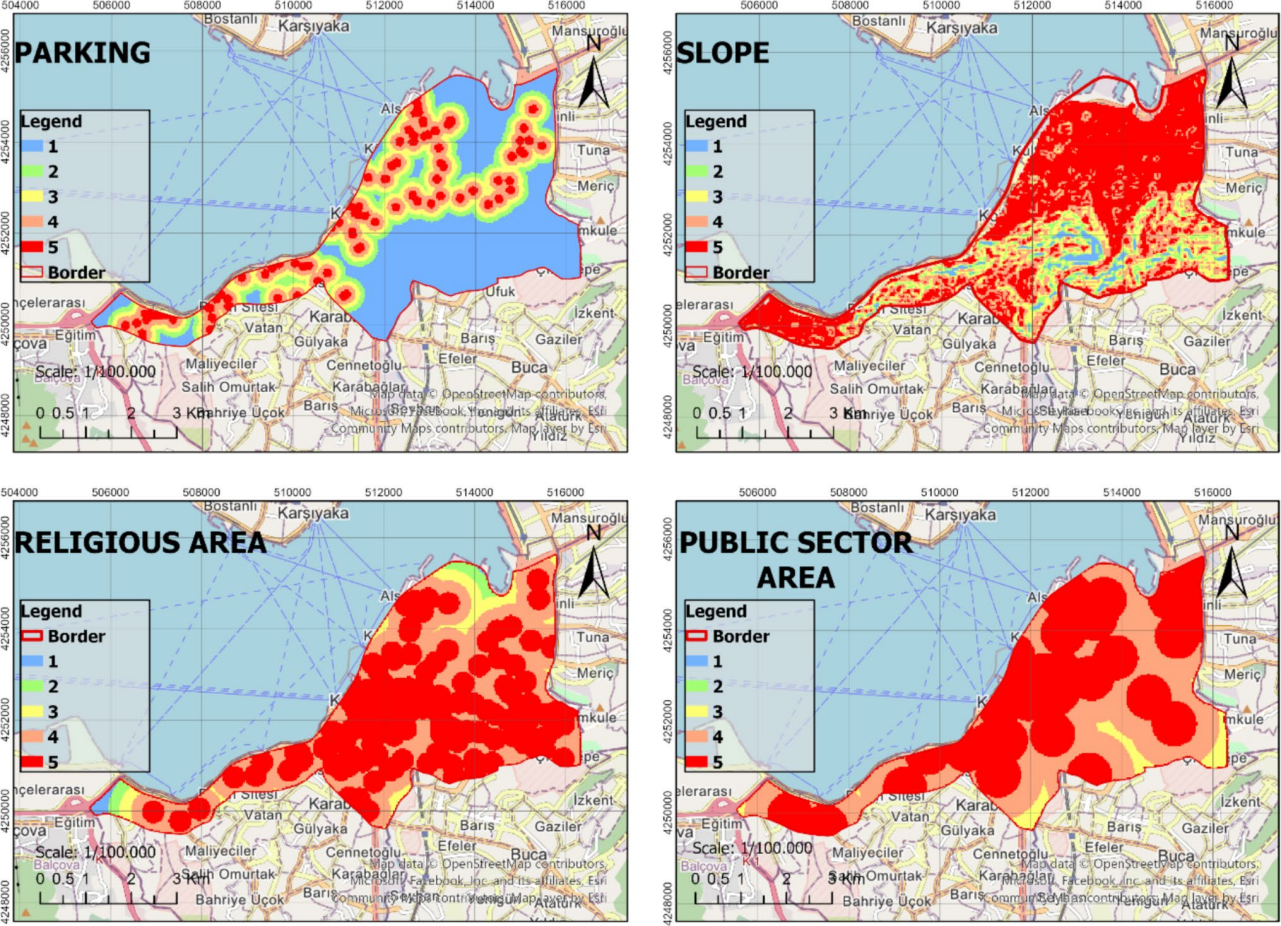
Suitable map (Criteria: 5–8)

**Fig. 6:**
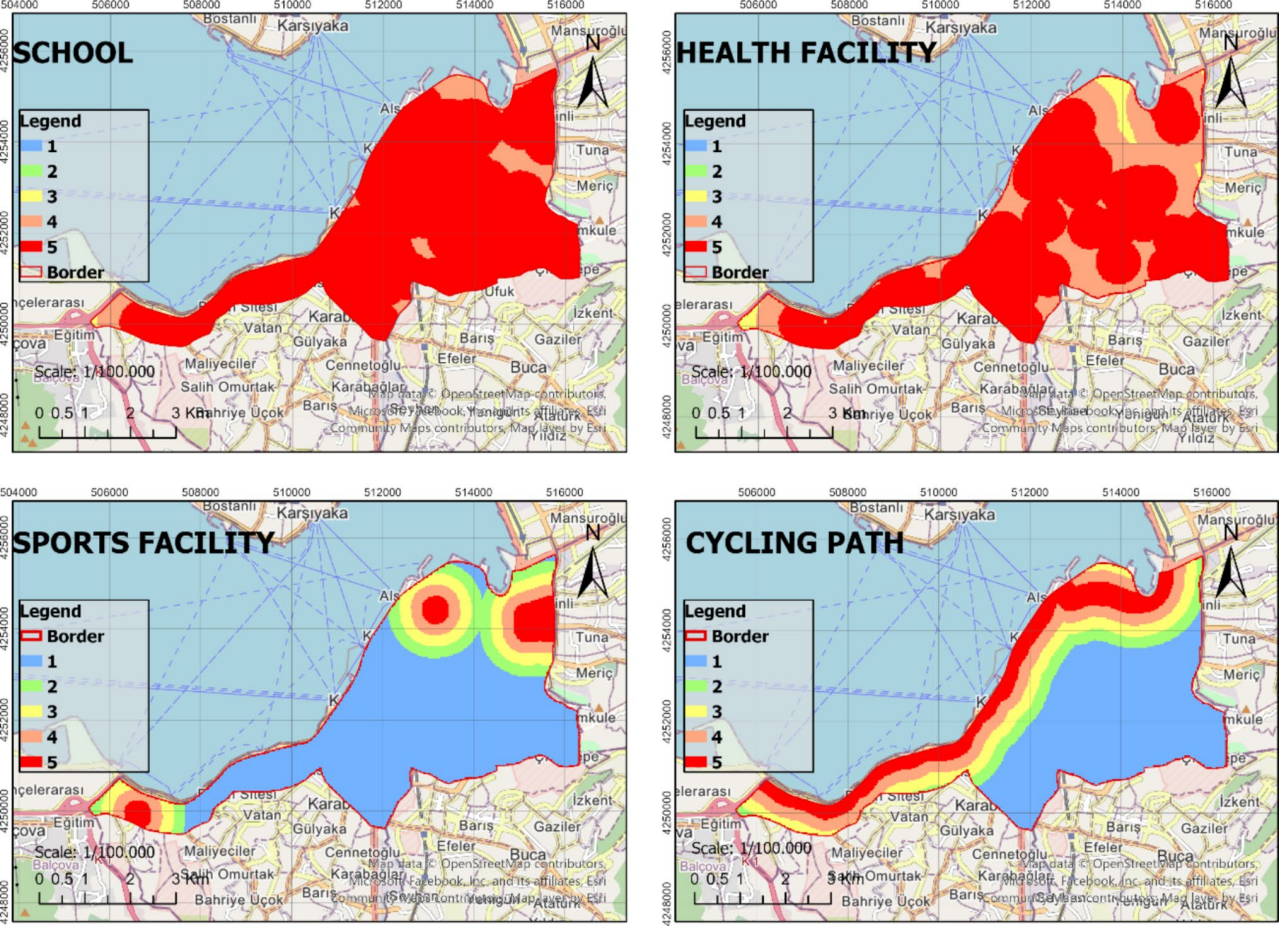
Suitable map (Criteria: 9–12)

**Fig. 7:**
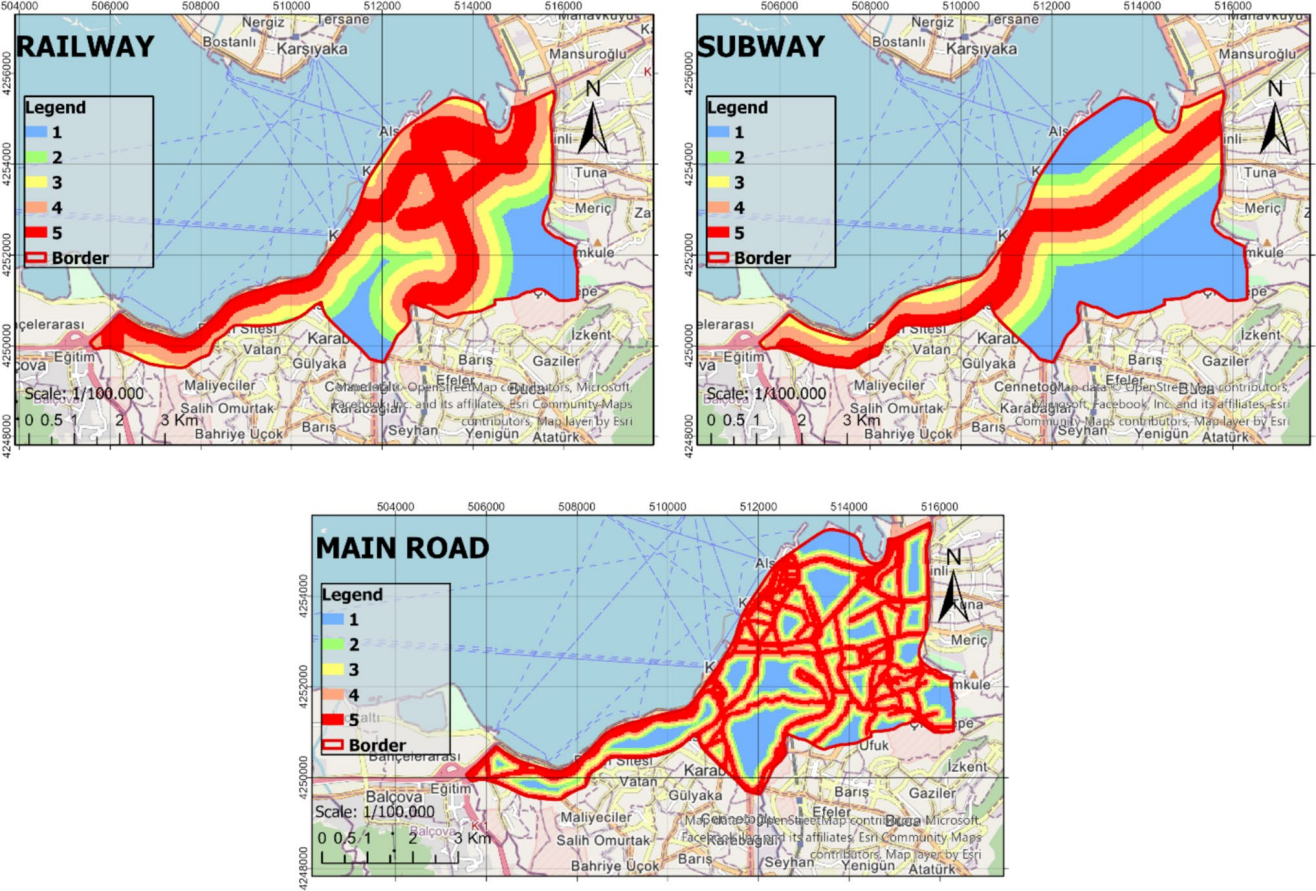
Suitable map (Criteria: 13–15)

In the process of identifying the most suitable green spaces, AHP, RF, and WLC methods were fully integrated with GIS. This integration ensured that the methods used in the decision-making process were congruous and complemented each other effectively. The identified criteria were thoroughly analyzed based on the sub-criteria presented in Table 2, thus resulting in the generation of raster data. The analysis of the raster data was conducted at a resolution of 30 m × 30 m, which enhanced the spatial accuracy of the study. The obtained raster data were reclassified using the ArcGIS Pro software to create suitability maps. These maps were rated such that areas with a score of 1 were considered unsuitable, while areas with a score of five were considered to be the most suitable locations (Figs. 4, 5, 6, and 7). In the classification of the suitability maps, the sub-class intervals specified in Table 2 were taken into account, thereby clearly illustrating the contribution of each sub-criterion in the evaluation process. This methodological approach provides an objective and systematic evaluation for the optimal selection of green spaces, thereby offering valuable contributions to planning and management processes.

**Fig. 8:**
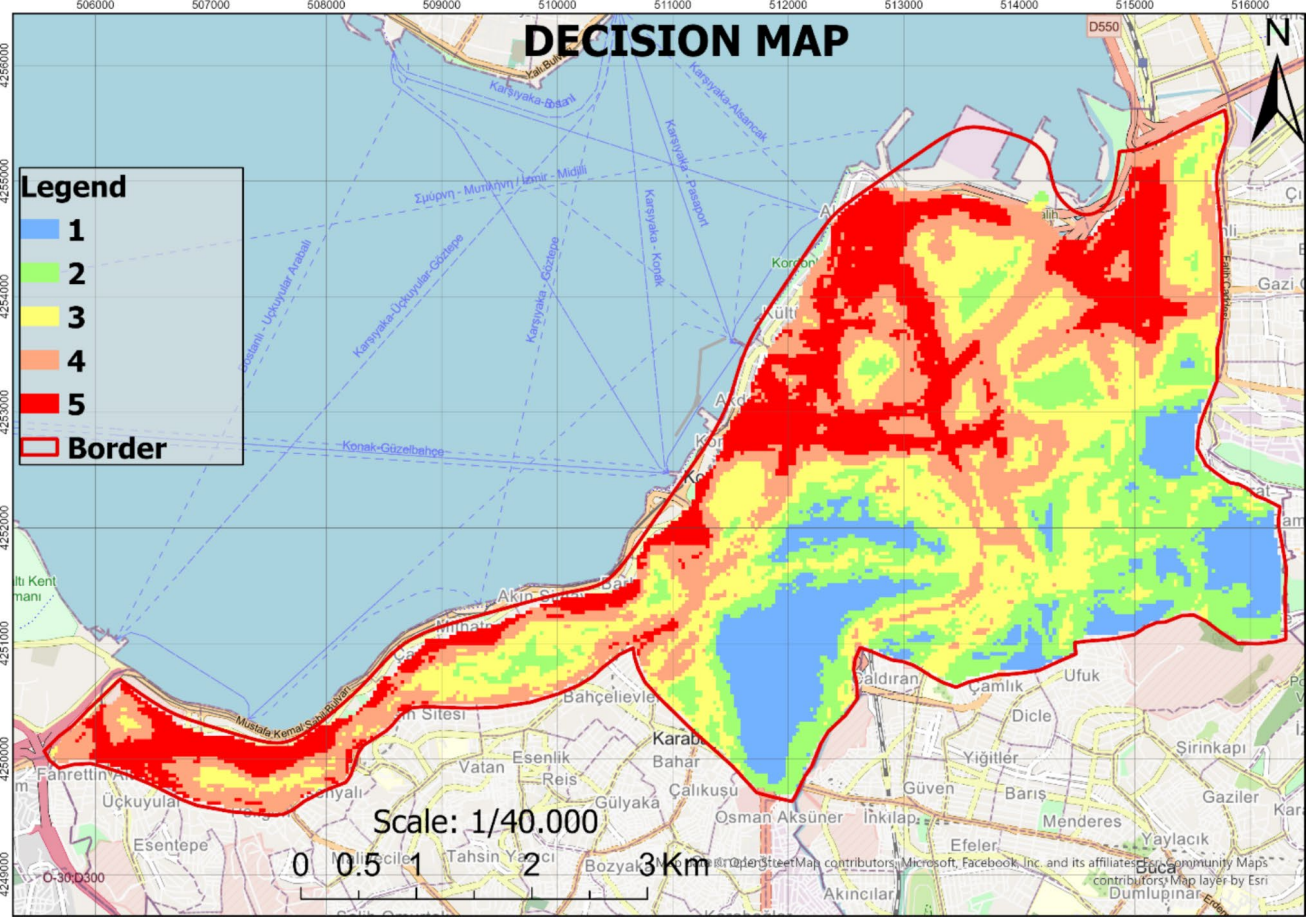
Decision map

Each data layer obtained through the aforementioned process was multiplied by the weights determined using the hybrid method. The weighted layers were then integrated using the WLC method. Consequently, decision maps with a cell resolution of 30 m × 30 m were generated. This operation was performed using the “Raster Calculator” tool in the ArcGIS Pro software (Fig. 8).

### The ranking of the most suitable areas using the TOPSIS method

**Fig. 9:**
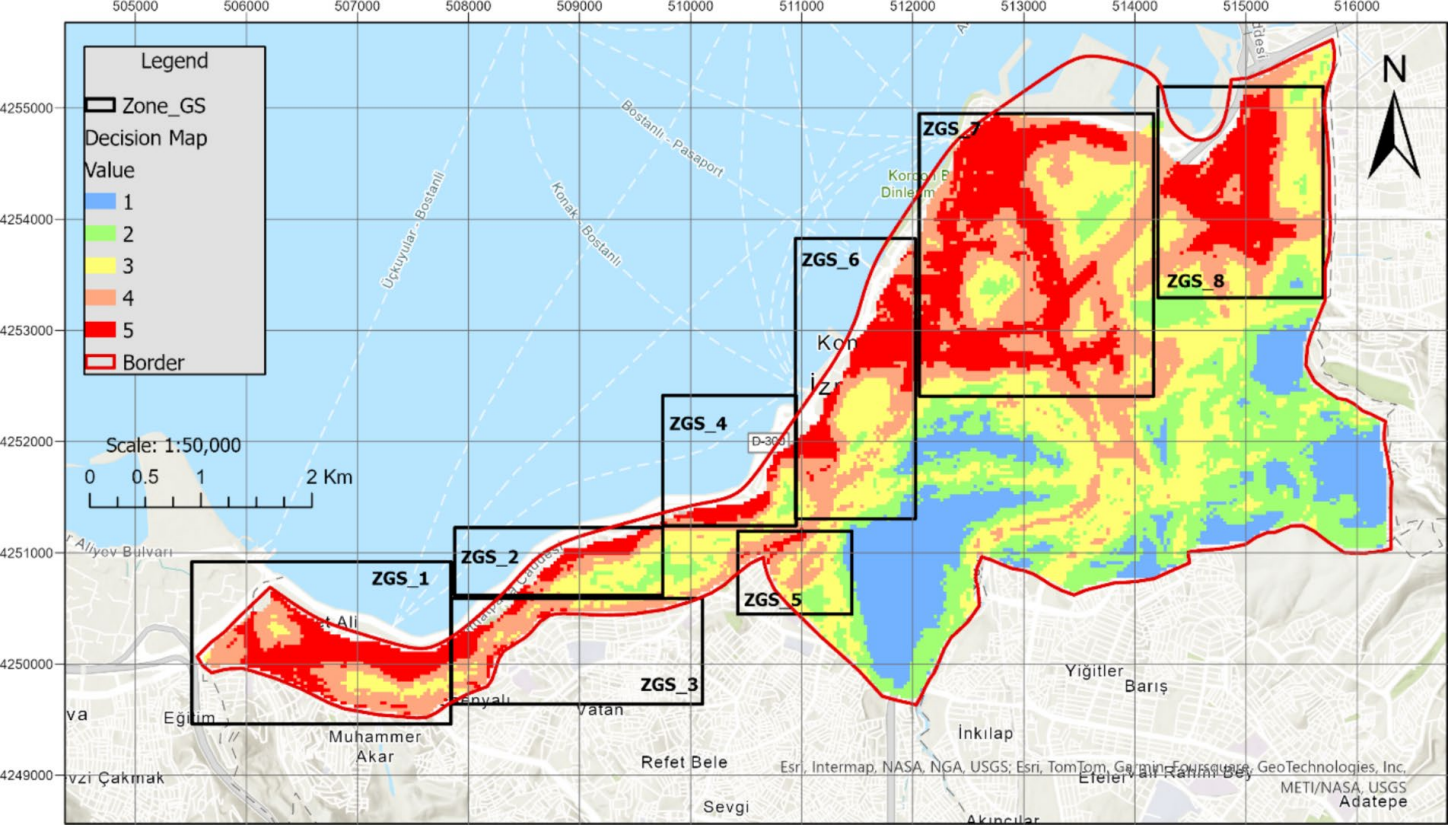
Alternative green space zones according to the most suitable areas

In this study, the most suitable areas for planning green spaces were identified (Fig. 8). However, prioritizing which specific regions within these suitable areas should be allocated for green spaces presents a significant challenge for decision-makers. To address this issue, the TOPSIS method was employed. Using the TOPSIS method, the pixel values corresponding to the alternative areas designated for each data layer (Fig. 9) were utilized to rank these areas (Table 7).

**Table 7:** The ranking of alternative zones via TOPSIS

Zone index	Si +	Si-	Ci	Rank
ZGS_1	0.0384	0.0308	0.4449	6
ZGS_2	0.0309	0.0433	0.5835	2
ZGS_3	0.0357	0.0432	0.5476	3
ZGS_4	0.0567	0.0363	0.3905	8
ZGS_5	0.0483	0.0469	0.4929	4
ZGS_6	0.0254	0.0569	0.6917	1
ZGS_7	0.0458	0.0352	0.4343	7
ZGS_8	0.0513	0.0426	0.4533	5

The TOPSIS method was chosen for ranking suitable green spaces due to its ability to provide a clear and interpretable decision-making framework based on ideal and anti-ideal solutions. Compared to other multi-criteria decision-making techniques such as VIKOR and ELECTRE, TOPSIS offers several advantages. For instance, VIKOR is highly sensitive to weight variations and requires additional compromise programming techniques, which may introduce uncertainty in practical applications, whereas ELECTRE is based on pairwise comparisons and preference relationships, making it computationally more complex and less adaptable to spatial decision problems. In contrast, TOPSIS effectively integrates multiple weighted criteria into a single ranking, while maintaining computational efficiency and interpretability. Hence, this makes it a more suitable approach for urban green space planning.

The ranking criteria used in the TOPSIS method were derived from the normalized hybrid method, which integrates AHP (expert-based weighting) and RF (data-driven weighting) to ensure a balanced and robust decision-making framework. This approach allows the ranking process to incorporate both subjective expert knowledge and objective machine learning-based weight distributions. Each criterion was normalized to maintain comparability across different units of measurement and ensure consistency in the evaluation process. The final ranking was determined based on the weighted distance calculation from the ideal and anti-ideal solutions to ensure that the most suitable locations align closely with optimal green space characteristics as defined by the hybrid weighting approach. This systematic methodology enhances the reproducibility of the ranking method and makes it adaptable to other urban environments facing similar green space planning challenges.

## Results

Upon analyzing the study’s results, it was observed that the suitability values calculated for each criterion based on its sub-criteria exhibited variations. It was determined that a hybrid approach employing AHP, RF, and WLC methods would lead to differences in the identification of suitable areas. The primary reason for this variation is that the criteria used receive different weights from the various methods applied. Thus, it was evaluated that reducing the criterion weights obtained from the AHP and RF methods to a single weight within the hybrid approach would be an effective strategy to minimize errors that may arise from methodological discrepancies. This integration ensures that criterion weights are consistently determined, which contributes to the generation of more reliable and robust results.

Furthermore, the ability to determine criterion weights using ML methods allows weight determination beyond traditional MCDM methods. This enhances the significance of the study, as the flexibility and adaptability of ML methods facilitate the accurate and objective determination of weights across different datasets and various conditions. The utilization of ML methods enables the dynamic adjustment of criterion weights, thereby enabling the development of more flexible and responsive approaches in decision-making processes.

The fusion of AHP, RF, and WLC methodologies ensures a more consistent and error-free integration of criterion weights, resulting in more reliable outcomes in the suitability assessment of green spaces within urban planning. This methodological approach provides significant advantages to decision-makers in achieving sustainability and livability goals for cities and provides valuable contributions to future planning processes.

Additionally, one of the most significant contributions of the TOPSIS method to this study is the transparent and systematic weighting of criteria used in the evaluation of alternatives. This method, through the integration of criterion weights determined by methods such as AHP and RF, clearly delineates the impact of each criterion on investment decisions. Consequently, it minimizes subjective errors in the prioritization process of investment areas. Moreover, the TOPSIS method makes substantial contributions to the budgeting process in the planning of green spaces from a public allocation perspective. Thereupon, by enabling the evaluation of alternatives based on economic and environmental criteria, the method facilitates the selection of the most efficient investment decisions under budget constraints. This ensures the effective use of public resources and enhances the sustainability of green space projects. Furthermore, the prioritization provided by TOPSIS is of great importance for making strategic decisions in future planning processes. Thus, this method is expected to support cities in achieving their sustainability objectives by contributing to the development of long-term plans to meet current and future green space needs.

### Evaluation of decision maps obtained through the new method

Upon examining the decision map generated using the hybrid method (Fig. 6), it was identified that the most suitable areas for green spaces (score 5) are predominantly located in the northern, northwestern, and western regions of the study area. Conversely, the unsuitable areas (score 1) are primarily situated in the southern and southeastern sections. These outcomes were greatly influenced by the prominence of the main road, railway, and slope criteria as the three most influential factors. Specifically, the most suitable areas (score 5) are characterized by minimal slope and proximity to major roads and railways. Additionally, it was observed that the suitability of other areas exhibits an increasing trend towards the eastern part of the study area, thus indicating a shift in weighted importance.

Furthermore, the locations of existing green spaces were analyzed in relation to the suitability results obtained. The analysis revealed that, out of 44 green spaces, only 11 are situated in the most suitable areas (score 5). Seventeen green spaces were found in the very suitable areas (score 4), while seven green spaces each are located in the suitable (score 3) and slightly suitable areas (score 2). Notably, only two green spaces fall within the unsuitable areas (score 1).

These findings highlight the critical influence of key criteria on the spatial distribution of green spaces and underscore the importance of strategic planning to enhance the placement of future green areas. By aligning existing green spaces with the identified suitability zones, urban planners can optimize the distribution and effectiveness of green infrastructure, thereby contributing to the overall sustainability and livability of the city.

### Evaluation of the ranking zones through the TOPSIS

As a result of the application of the TOPSIS methodology, the ZGS_6 area was identified as the primary region for initial green space planning, while the ZGS_4 area was prioritized last. The analysis of the locations of existing green spaces has revealed a predominant concentration in the western and southeastern regions. In contrast, the ZGS_7 and ZGS_8 areas each contain only three green spaces. This finding demonstrates that the weighted criteria significantly influence the selection of location regions.

Furthermore, it has become evident that policymakers and local government administrators need to correlate the results obtained to the existing infrastructure. Thus, the establishment of this correlation will facilitate needs assessments, thereby enabling the effective preparation of investment programs. This alignment ensures that green space development is strategically integrated with existing infrastructural frameworks that promote sustainable and efficient urban planning.

## Discussion

This study presents an innovative model for sustainable urban planning by integrating AHP, WLC, and TOPSIS methods with ML and GIS to evaluate the suitability of green spaces in the Konak district of İzmir. Compared to previous studies in the literature, the proposed approach demonstrates notable advancements in both application and analytical rigor, and it offers significant methodological and practical contributions.

In the literature on urban green space planning, MCDM and GIS methods are widely utilized. For instance, Dimitrova and Dzhambov (2017) focused on spatial analyses to evaluate the environmental impacts of green spaces, such as accessibility and air quality. However, their study does not incorporate ML methods that enable dynamic adaptation of criteria weights. Similarly, Liu and Russo (2021) employed a comprehensive set of criteria to assess the contribution of green infrastructure to ecosystem services, but their assessment was limited in terms of spatial accuracy and integration. Conversely, this study addresses these gaps by offering a more holistic approach that combines GIS-based analyses that ensure spatial accuracy with the flexibility of ML methods. Thus, our study employs an innovative hybrid method that integrates ML with the AHP.

Similarly, the study conducted by Ustaoglu and Aydınoglu (2020) analyzed land suitability in the Pendik district by using a combination of AHP, fuzzy set modeling, and GIS integration. Both studies highlight the potential of spatial decision support systems for contributing to sustainable urban planning. However, the incorporation of ML methods and the diversity of methodologies used in our research establish a novel framework that offers significant contributions to the literature.

Li et al. (2018) combined the AHP and contingent valuation (CV) methods in green space planning for Fuping County, China, to balance subjective and objective weights in suitability assessments. Similar to our study, both research studies emphasize the use of spatial decision-making techniques to evaluate green space suitability. However, our research distinguishes itself by enabling dynamic data analysis and offering enhanced spatial resolution by employing a hybrid approach that integrates the AHP with the RF algorithm.

Abebe and Megento (2017) utilized GIS-based MCDM methods to identify suitable areas for urban green spaces in Addis Ababa. While both studies emphasize the importance of spatial analyses in green space planning, our research integrates the RF algorithm to offer a more dynamic and adaptable model that is tailored to local social demands. Unlike Abebe and Megento, who primarily focused on physical and spatial factors such as slope, soil type, and proximity to roads, our study incorporates criteria such as parking, bike sharing stations, religious areas, and sports facilities, thereby directly addressing user needs and social demands. These distinctions render our approach more inclusive and applicable within a broader framework.

Another significant contribution of our study is the development of an investment prioritization system aimed at maximizing the efficient use of public resources. A critical advantage in achieving sustainable urban development goals is the TOPSIS method’s ability to provide a balanced evaluation of various criteria, which allows the strategic allocation of public investments. Thus, by integrating machine learning techniques with TOPSIS for investment area prioritization and location selection, our study presents a novel hybrid approach that provides a more advanced and adaptable framework.

Furthermore, urban green space management was approached as a “comprehensive framework” in the study by Jansson and Lindgren (2012), which emphasized the need to consider social, economic, and ecological dimensions together. By incorporating more technical tools, our study expands on this framework and offers a model that can more accurately and dynamically address user demands. For instance, in addition to GIS-based spatial analyses, the integration of the RF algorithm has introduced greater flexibility and accuracy to the decision-making process. This feature aligns with the participatory and strategic approach proposed by Gustavsson et al. (2005) for urban forest management, although such models often lack dynamic data analysis capabilities. Furthermore, Gustavsson et al. (2005) highlighted the importance of GIS-based analyses in urban planning. However, our study advances these traditional GIS approaches by integrating ML algorithms to create a system that can adapt to changing urban dynamics swiftly.

Bai et al.’s (2022) study in Fengdong New City used GIS and remote sensing (RS) to develop ecological corridors, and they employed a BP neural network to weight the criteria. In contrast, our research presents a model that integrates AHP, WLC, TOPSIS, and the RF algorithm. Although both studies leverage GIS and data-driven criteria weighting methods as common tools to enhance sustainable urban planning and green space management, our approach distinguishes itself by combining AHP with the RF algorithm, which offers more dynamic adaptability. Furthermore, our study provides more detailed analyses with a spatial resolution of 30 × 30 m, which contributes to higher precision in decision-making.

Yang et al. (2024) analyzed the nonlinear and interactive relationships between active transportation behaviors and green spaces in Chengdu using Strava data and SHAP algorithms. While both studies adopt data-driven approaches, our research provides a more inclusive framework by incorporating local user demands into the analysis. Additionally, Yang et al.’s study introduces new dimensions through dual-perspective measurements, such as street-level and aerial evaluations of green spaces. While our study focuses on spatial resolution and strategic management, their research examines the ecological and social impacts of active transportation at a more micro level. These distinctions highlight the complementary nature of both approaches, thus offering unique contributions to sustainable urban planning and green space management.

Within the context of these comparisons, certain limitations must also be acknowledged. Our study does not incorporate social and behavioral data. Methods such as Public Participation GIS (PPGIS), as utilized in studies like Ives et al. (2017), provide tools that can more directly represent community needs and values. Additionally, the inclusion of seasonal and multi-temporal analyses could enable a broader evaluation of the ecological and sociocultural values of green spaces. In the future, testing the methodology in different cities to assess its generalizability and integrating social data could contribute to making the approach more comprehensive.

The findings of this study indicate that 75% of the existing urban green spaces in Konak are located in areas classified as low suitability zones. To validate these results, official municipal planning documents and academic studies were reviewed. The 2025–2029 Strategic Plan of Konak Municipality highlights the necessity of expanding and maintaining green spaces while addressing spatial inequalities in urban infrastructure. The report specifically emphasizes that current green areas are insufficient in terms of accessibility, ecological balance, and equitable distribution across the district. Furthermore, it states that green space development should align with a sustainability-driven urban strategy, which reinforces the need for optimized site selection (Konak Municipality 2024).

Additionally, an academic study conducted at İzmir Kâtip Çelebi University has identified deficiencies in the ecological and social functions of green spaces in Konak. For instance, the study emphasizes that existing green spaces do not fully align with the principles of urban sustainability and green infrastructure planning (Gözsoy 2024). These findings strongly correlate with the results of our hybrid AHP-ML-GIS methodology, which determined that the most suitable areas for new green spaces differ significantly from current allocations.

The consistency between the model outputs and real-world urban planning concerns further strengthens the validity of the proposed methodology. Given that both official reports and independent academic research highlight the misalignment of existing green spaces with optimal locations, the decision-support framework developed in this study offers a scientifically grounded and policy-relevant approach to improving future green space planning.

In conclusion, this study presents an innovative framework for urban green space planning by integrating MCDM, ML, and GIS methods. This hybrid approach addresses the shortcomings of existing methods in the literature by offering a robust model for sustainable urban planning. In the future, optimizing the framework for broader applications by incorporating methods that enhance social participation will further amplify the impact of our study.

## Conclusion

This study used the Konak district of Izmir as a case study to assess the suitability of green spaces in urban planning using a hybrid approach that integrated the AHP, RF, and WLC methods with the TOPSIS methodology. The results indicate that different methodologies influence criterion weights in varying ways; however, these discrepancies are minimized through the hybrid approach. By synthesizing the weights obtained from AHP and RF through the WLC method, inconsistencies in individual methodologies are effectively reduced. This thus leads to more consistent and reliable outcomes in green space suitability assessment.

The integration of ML methods into the weighting process extends beyond traditional MCDM techniques and offers a more data-driven and adaptive methodology. The results demonstrate that ML methods provide a more flexible and objective approach to determining criterion weights across diverse datasets and varying conditions. Among the ML models applied, the RF model exhibited the highest predictive performance, achieving an accuracy of 0.69, precision of 0.70, recall of 0.69, F1-score of 0.68, and an area under the curve (AUC) value of 0.90. Given its robust performance, RF was incorporated into the hybrid approach to enhance the reliability of the weighting process.

The TOPSIS methodology was employed to prioritize alternative green space investment areas objectively, which made sure that the impact of each criterion on spatial decision-making was clearly delineated. This method proved particularly valuable in budgeting and public resource allocation, as it facilitated the selection of investment areas based on economic, environmental, and spatial suitability factors. The application of TOPSIS in conjunction with hybrid weighting enhances the sustainability and efficiency of decision-making in urban green space planning by minimizing subjective errors in the prioritization process.

An analysis of the decision map generated through the hybrid approach (Fig. 8) indicates that the most suitable and highly suitable areas are concentrated in the northern and western parts of the study area. This spatial distribution is influenced by key factors such as minimal slope, proximity to major roads and railway networks, and accessibility to public services. Conversely, the unsuitable areas are primarily located in the southern and southeastern regions, where steep slopes, lack of transportation infrastructure, and dense urbanization limit the feasibility of green space development.

Further analysis of existing green spaces revealed that a significant portion (75%) of these areas are not optimally distributed in relation to suitability zones. Among the 44 existing green spaces, only 11 are located in the most suitable areas (score 5), while 17 are in the very suitable areas (score 4), seven in the suitable areas (score 3), seven in the slightly suitable areas (score 2), and two in the unsuitable areas (score 1). This distribution highlights critical urban planning deficiencies and emphasizes the need for strategic interventions to ensure that green spaces are aligned with high-suitability locations. Several factors contribute to this misalignment, including historical urban expansion patterns, inadequate spatial planning regulations, and socio-economic disparities that influence land use decisions. The concentration of green spaces in less suitable areas can be attributed to legacy planning decisions, where land availability rather than strategic suitability dictated green space allocation. Additionally, economic constraints, land ownership issues, and conflicting land-use priorities may have further restricted the optimal placement of green spaces. Future urban planning strategies should address these systemic challenges by prioritizing suitability-based green space development, integrating socio-economic considerations, and adopting adaptive planning policies that respond to evolving urban dynamics.

The findings of this study demonstrate that the integration of MCDM, ML, and GIS methodologies can significantly enhance urban green space planning by providing a more structured, data-driven, and scientifically robust approach. The hybrid approach ensures that criterion weight inconsistencies are minimized, while the TOPSIS ranking system facilitates effective resource allocation. This methodological integration offers substantial advantages for long-term urban sustainability, particularly in optimizing land use and maximizing environmental benefits.

In addition, this study provides insightful information to policymakers, planners, and decision-makers who aim to improve green space planning and allocation strategies in urban areas. To ensure that urban green spaces are optimally distributed, it is recommended that data-driven GIS-based decision support systems be integrated into municipal planning processes. Furthermore, policymakers should prioritize the development of green spaces in high-suitability areas while also considering the rehabilitation or relocation of existing green areas that are currently misaligned with optimal locations.

Additionally, public–private partnerships can be leveraged to facilitate new green space development projects and ensure that investments are directed toward the most environmentally and socially beneficial locations. Future urban development strategies should also emphasize the integration of green infrastructure with transportation and public services, thus allowing for more accessible and multifunctional green spaces. Furthermore, urban planning should take climate resilience into account by promoting the establishment of ecological corridors and nature-based solutions that enhance environmental sustainability and urban livability. By adopting a systematic, data-driven, and sustainability-oriented approach, policymakers can ensure that urban green spaces contribute to ecological balance, public health, and climate adaptation, all of which will ultimately enhance the quality of life of urban residents.

For future research, applying this hybrid methodology to different cities could help assess the generalizability and adaptability of the approach in diverse urban contexts. Additionally, incorporating additional MCDM methods and advanced artificial intelligence techniques could further refine green space planning strategies. The limitations of this study include its reliance on specific criteria and regional constraints, which may not fully capture the complexities of green space planning in different urban settings. Hence, expanding the dataset to include a broader range of socio-economic, demographic, and ecological variables would improve the applicability and scalability of the model.

In conclusion, this study presents a comprehensive and adaptable methodological framework for optimizing urban green space allocation. The integration of AHP, RF, WLC, and TOPSIS provides urban planners and policymakers with a transparent, data-driven, and objective decision-support system that can contribute to the development of sustainable, equitable, and livable urban environments.

## Data Availability

Upon request, all data and materials utilized in this study will be made readily accessible.
